# Expanding the differential diagnosis of bone-forming tumors: a new entity characterized by a *NIPBL::BEND2* fusion

**DOI:** 10.1007/s00256-025-05109-8

**Published:** 2025-12-26

**Authors:** Danielle Forster, Rashed Al-Khudairi, Fiona Bonar, Alison Cheah, Wendy Brown, Richard Boyle, Dorte Wren, Adrienne Flanagan, Fernanda Amary, Paul O’Donnell

**Affiliations:** 1 Department of Radiology, Royal National Orthopaedic Hospital, Stanmore, UK; 2 Department of Anatomical Pathology, Douglass Hanly Moir Pathology, Sydney, Australia; 3 Department of Radiology, Royal Prince Alfred Hospital, Sydney, Australia; 4 Department of Orthopaedics, Royal Prince Alfred Hospital, Sydney, Australia; 5 NHS North Thames Genomic Laboratory Hub, Great Ormond Street Hospital for Children NHS Foundation Trust, London, UK; 6 Department of Pathology, Royal National Orthopaedic Hospital, Stanmore, UK; 7 Research Department of Pathology, Cancer Institute, University College London, London, UK

**Keywords:** Bone-forming tumors, Osteoid-producing tumors, Osteosarcoma, Osteoblastoma, Osteoma, Bone neoplasms, Musculoskeletal radiology, Diagnostic imaging, CT, MRI, Radiographic features, Bone tumor classification, Bone matrix production, Skeletal oncology, Gene fusion, Molecular profiling, NIPBL::BEND 2 Fusion, Next-Generation Sequencing (NGS), Skeletal radiology, Osteogenic neoplasm

## Abstract

We report two bone-forming tumors driven by a newly recognized fusion. The first is a lesion in the distal radius of a 59-year-old female subject, which had been present for 10 years. Imaging showed an intramedullary tumor with an exophytic dorsal component and marked sclerosis, histologically best classified as low-grade osteosarcoma. The second was a destructive lesion in the left ulna of a 13-year-old female with a short history of elbow pain. Imaging showed a lytic lesion with subtle tumor ossification, histologically best classified as an atypical osteoblastoma-like tumor. A *NIPBL::BEND2* fusion was found in both cases on whole genome sequencing. This fusion has been previously reported in three bone lesions, two of which were reported as phosphaturic mesenchymal tumors and the other exhibited features of a high-grade osteosarcoma. These newly reported cases were bone-forming, but histologically indolent, with no features of high-grade osteosarcoma, and did not demonstrate the typical features of a phosphaturic mesenchymal tumor nor evidence of tumor-induced osteomalacia. The World Health Organization has not established a specific name for tumors associated with a *NIPBL::BEND2* fusion, and their biological potential cannot currently be predicted. These cases add to the expanding clinical and histological knowledge of this entity and illustrate that, in addition to the other rarely reported phenotypes, low-grade bone-forming tumors may also be caused by this exceptionally rare fusion.

## Introduction

A primary bone tumor with a *NIPBL::BEND2* fusion was first reported in 2023 [1] in a 12-year-old male who presented with a mass in the left fibula, which showed expansile remodeling; there were clinical and radiographic features of tumor-induced osteomalacia, and serum fibroblast growth factor 23 (FGF23) was markedly elevated. An osteoblastoma-like phosphaturic mesenchymal tumor (PMT) was diagnosed, and after resection of the lesion, FGF23 and hypophosphatemia normalized, the former within hours of surgery. RNA sequencing of the resection specimen identified a novel *NIPBL::BEND2* fusion.

Subsequently, RNA sequencing of 76 cases of PMT found *FN1::FGFR1* to be the most common genetic alteration (56.6% of cases) [2]. Among a small number of other novel rearrangements, a *NIPBL::BEND2* fusion was detected in a single case, associated with a tumor in the ilium and tumor-induced osteomalacia.

More recently, a bone-forming tumor showing a *NIPBL::BEND2* fusion was reported in the third metatarsal of a 17-year-old male [3]. This case, discovered incidentally after trauma, showed indolent clinical and radiologic features and was thought to represent a benign osteoblastic lesion. However, after repeated local recurrences, the final histology resembled a high-grade osteosarcoma.

We report two additional tumors with a *NIPBL::BEND2* fusion to further illustrate the heterogeneity of this lesion. Both were bone-forming tumors but clinically and histologically distinct, exhibiting no high-grade histological appearances, clinical or imaging features of PMT, nor evidence of tumor-induced osteomalacia.

## Case presentations

### Case 1

**Fig. 1:**
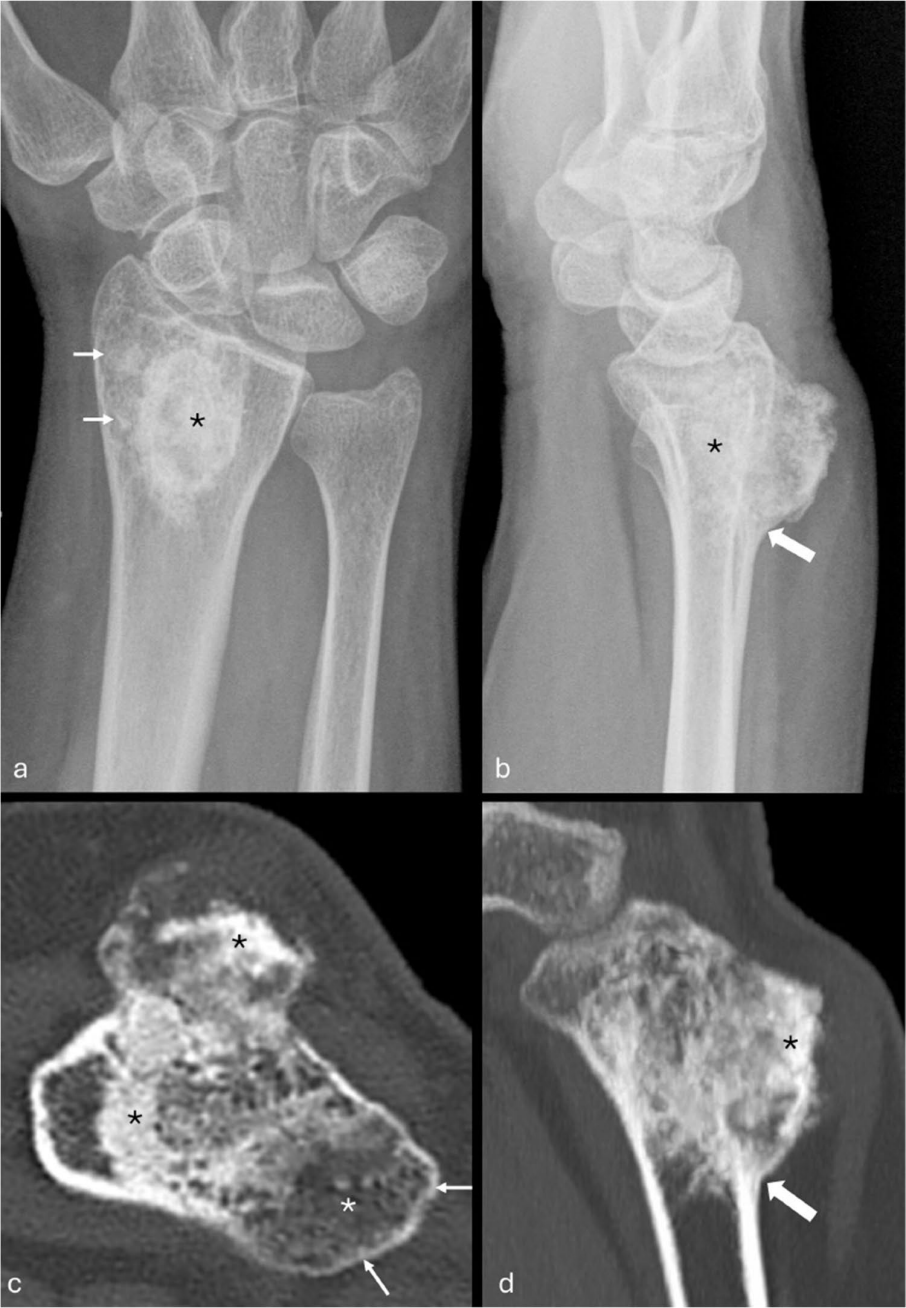
Case 1. Frontal (**a**) and lateral (**b**) radiographs of the right wrist. A well-defined focus of increased density is projected over the distal radius (asterisk, **a**) with further faint densities laterally (arrows). A heavily ossified mass protrudes dorsally, but increased density projected over the distal radius suggests intramedullary tumor (asterisk, **b**). The dorsal cortex is “split” at the proximal aspect of the tumor suggesting intracortical growth (block arrow, **b**). Axial (**c**) and sagittal (**d**) CT reconstructions of the right wrist. There is heterogeneous intramedullary sclerosis and an ossified exophytic mass (black asterisks). Tumor at the radial/lateral aspect of the radius shows only faint sclerosis (white asterisk, **c**) and there is adjacent cortical thinning (thin arrows, **c**). Sclerosis extends from the bone into the protuberant mass, and there is intracortical growth proximally (block arrow, **d**)

**Fig. 2:**
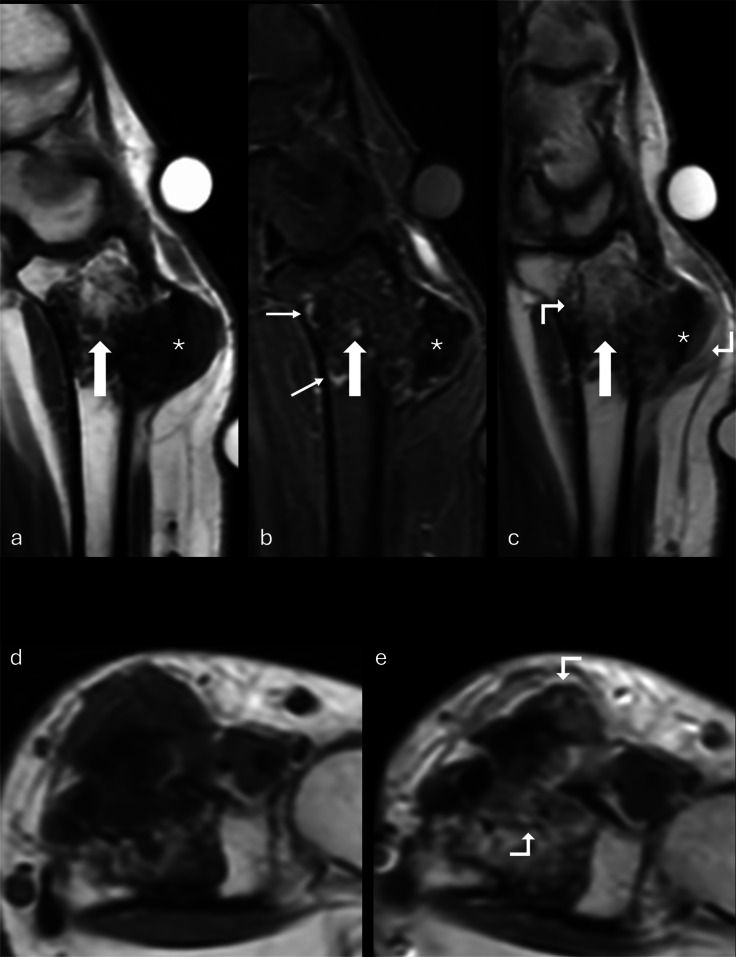
MR images of the right wrist mass. T1-weighted (**a**), STIR (**b**), and T1-weighted post-contrast (**c**) sagittal images; T1-weighted pre- (**d**) and post-contrast (**e**) axial images. Relatively homogeneous low signal intensity is seen in the ossified exophytic mass (asterisk, **a**–**c**); lobular intramedullary tumor reveals patchy low signal (block arrow), merging with fatty marrow and poorly distinguished on STIR images (**b**). Thin peripheral high signal intensity is seen (thin arrows, **b**). Mild enhancement of the intramedullary and predominantly the superficial aspect of the protuberant mass is seen (bent arrows, **c**, **e**)

A 59-year-old female presented with a 10-year history of a lump on the dorsum of the right wrist which had been slowly enlarging over the past year and causing intermittent pain. The patient gave no significant past medical history, family history, or history of trauma. The initial radiographs demonstrated a heavily ossified lesion in the distal radius, with a sessile exophytic mass projecting dorsally (Fig. 1a, b). CT confirmed diffuse sclerosis in the intra-osseous and exophytic components of the mass and intra-cortical growth proximally (Fig. 1c, d). Foci of fatty marrow and low signal (ossified) tumor were visible within the intramedullary component on MRI, with more confluent hypointensity suggesting ossification in the protuberant dorsal mass; there was minimal enhancement following intravenous Gadolinium administration (Fig. 2). No radiographic evidence of osteopenia was identified; serum calcium and phosphate were both normal, but FGF23 levels were not measured. The intramedullary location of the lesion excluded the diagnosis of an osteochondroma. A benign or low-grade malignant bone-forming lesion was considered, but of note, there was no adjacent marrow edema-like signal to support the diagnosis of osteoblastoma. After a non-diagnostic CT-guided needle biopsy, an open biopsy was performed, followed by resection and distant staging, with no evidence of metastatic disease on whole body MRI or CT chest.

**Fig. 3:**
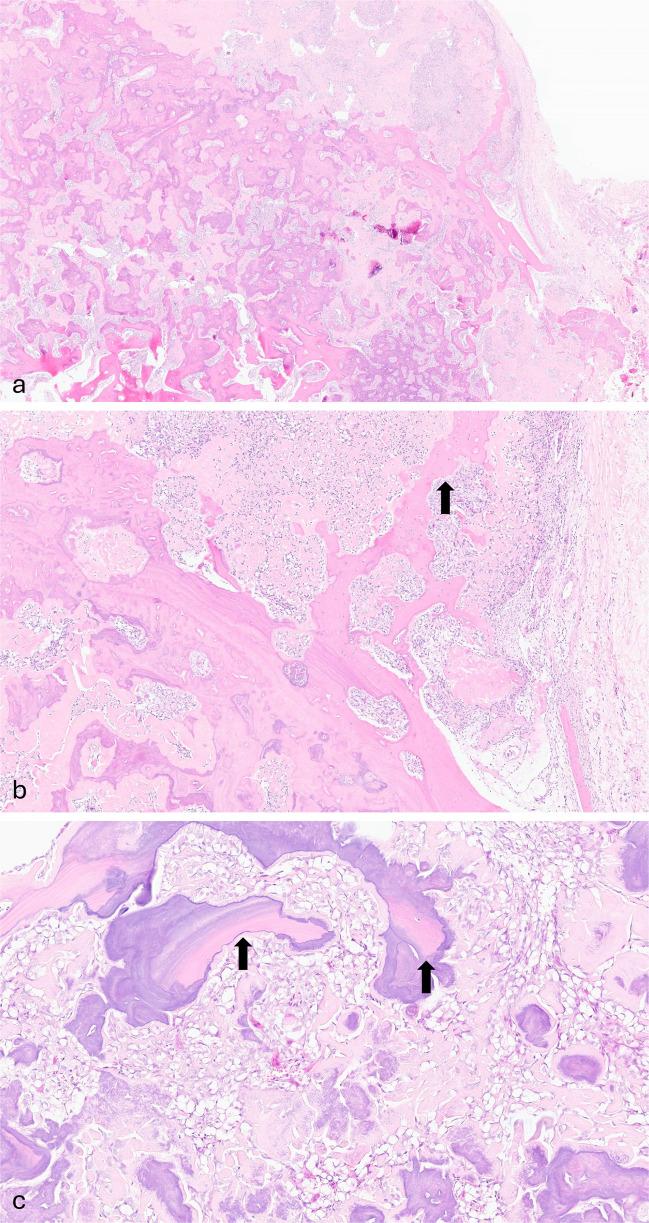

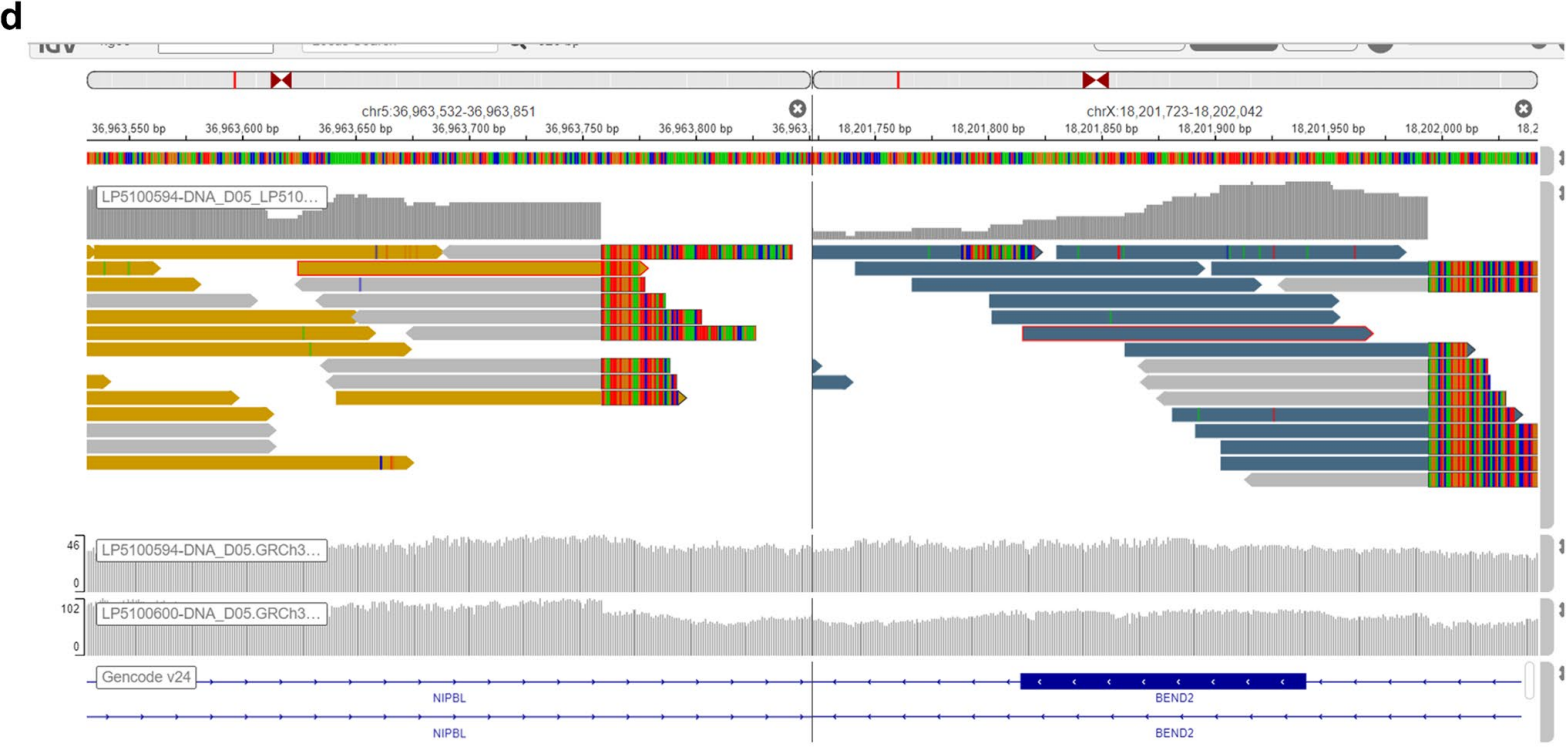
Microphotography of the bone-forming tumor with pre-existing lamellar bone entrapment (black arrows, **b** and **c**). **d** Genomic reads demonstrating the *NIPBL::BEND2* fusion

Tissue from both the open biopsy and the resection showed a bone-forming tumor composed of broad anastomosing trabeculae of woven bone lined by mildly atypical osteoblastic cells. There was permeation of the pre-existing bone, and the tumor extended into surrounding soft tissue (Fig. 3). There were few mitoses, and there was no tumor necrosis. Very occasional cells expressed c-fos*;* H3.3 G34W expression was not identified, and there was no evidence of *MDM2* gene amplification, effectively excluding giant cell tumor of bone and low-grade central osteosarcoma, respectively.

As the tumor was atypical for conventional osteosarcoma and surface bone forming tumors on both imaging and histology, whole genome sequencing was performed. The tumor was considered difficult to classify but showed a “quiet”/diploid genome without the many structural variants expected in a conventional osteosarcoma. An in-frame *NIPBL::BEND2* fusion was identified (Fig. 3d).

14 month radiographic and MRI follow-up of the right wrist demonstrated no evidence of local recurrence or pulmonary metastases on CT chest.

### Case 2

**Fig. 4:**
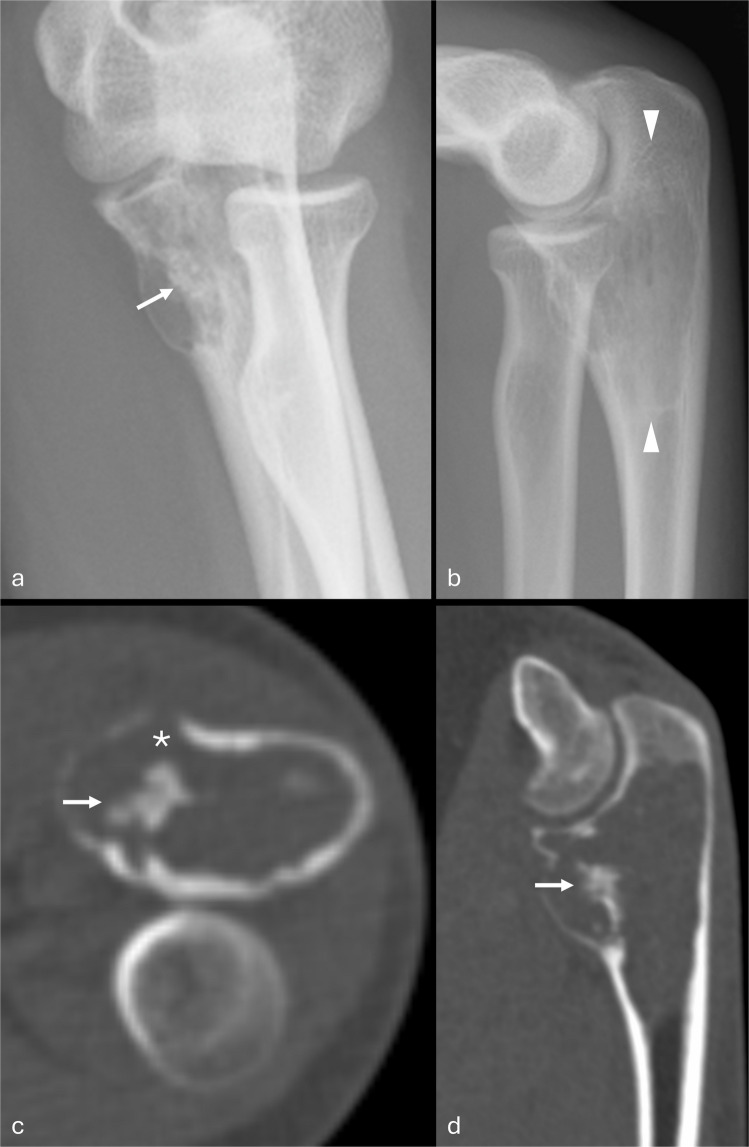
Case 2. AP (**a**) and lateral (**b**) radiographs of the left elbow. A lucent lesion is seen in the proximal ulna. There is mild expansile remodeling, and the anterior cortex is markedly thinned. A focus of increased density is identified (arrow, **a**), not seen on the lateral radiograph. The lesion is poorly defined (arrowheads, **b**) and slightly lobular. Axial (**c**) and sagittal (**d**) CT reformats show a focus of ossification in the anterior aspect of the lesion (arrows), marked thinning of the anterior cortex, and a small cortical breach (asterisk, **c**). There is lobular morphology with endosteal scalloping (**d**)

**Fig. 5:**
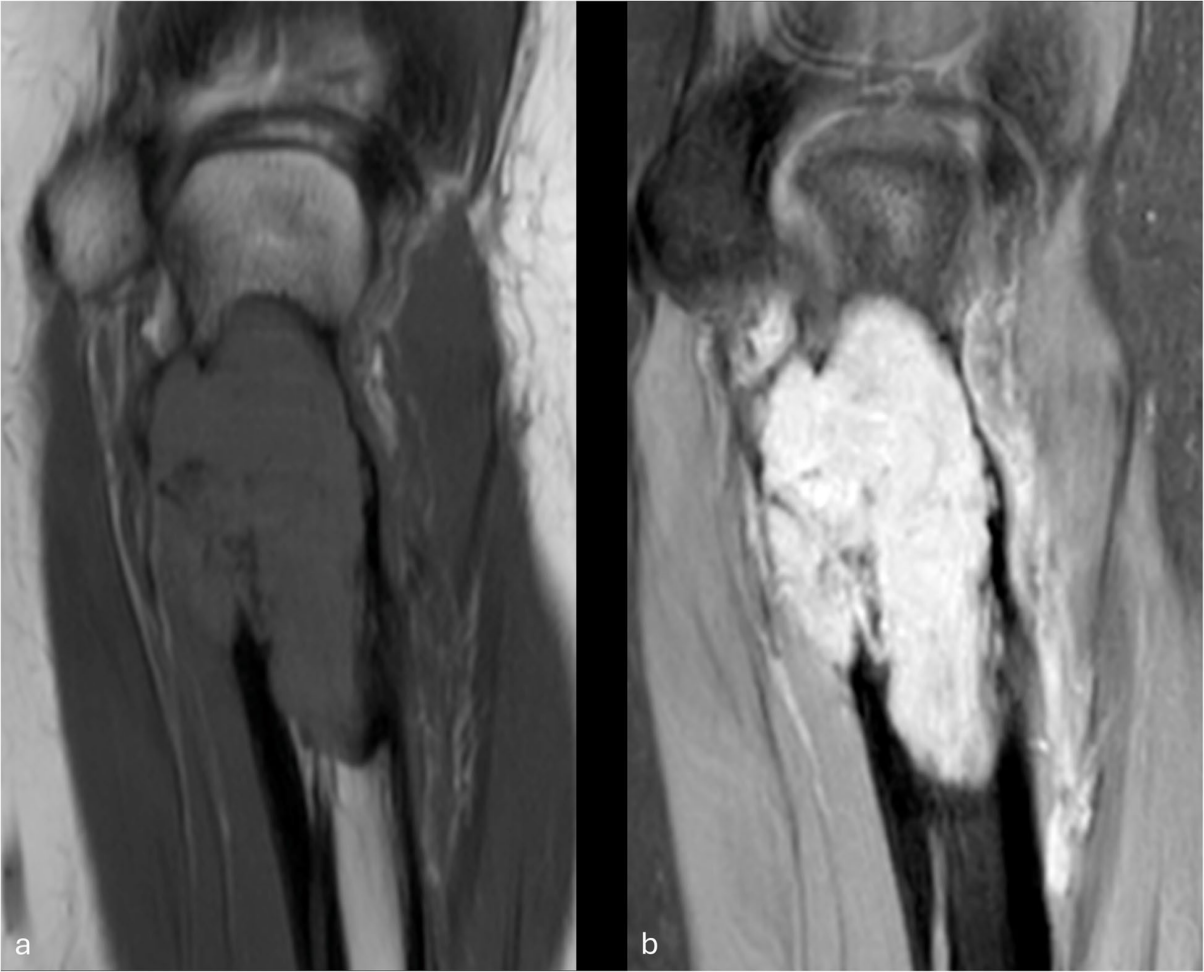

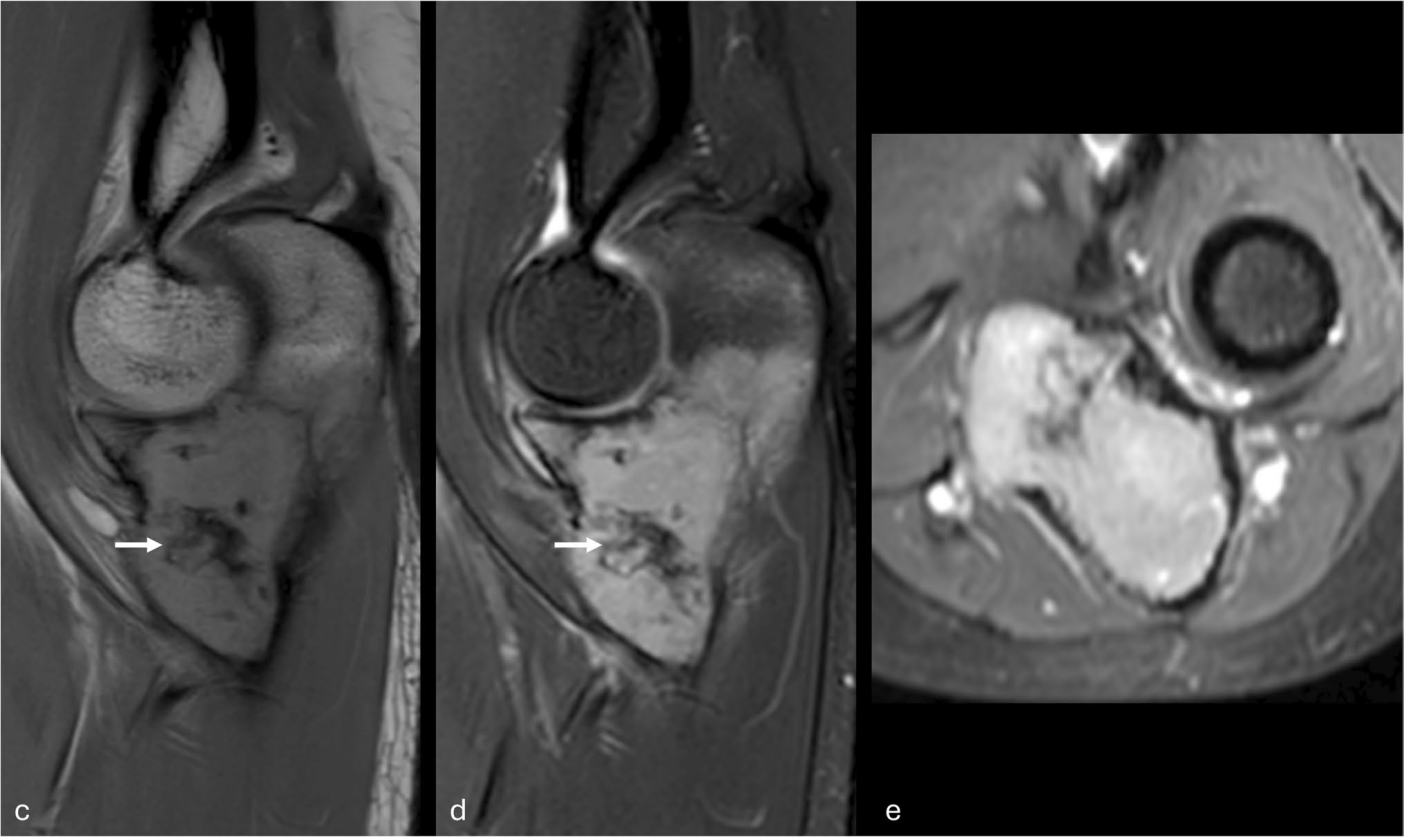
MR images of the left proximal ulna. Coronal T1 (**a**) and proton density (PD) fat saturated (FS) images (**b**) show a well-defined, expansile lesion which is mildly hyperintense on T1-weighted and markedly hyperintense on PDFS scans. Sagittal PD (**c**) and T2FS images (**d**) show a moderately hyperintense lesion extending to the subarticular bone, with a prominent central area (arrows) and small punctate foci of low signal intensity consistent with calcification. Axial T1-weighted FS post-contrast (**e**) image shows a diffusely enhancing lesion with eccentric low signal anteriorly due to calcification. The anterior cortex is markedly thinned, but no soft tissue extension is seen

A 13-year-old female presented with a 2-month history of pain in the left (non-dominant) elbow. Symptoms were mild at rest, but worse on activity or if there was minor contact with the ulna. There had been no history of trauma and there was no relevant past medical history. On examination, the elbow showed a full range of movement but there was tenderness and mild prominence of the medial aspect of the elbow. Radiographs showed a lucent lesion extending to the subarticular bone of the proximal ulna (Fig. 4a, b). The cortex was markedly thinned but intact and there was a focus of irregular density suggesting ossification within the lesion. Eccentric expansion of the anterior aspect of the ulna was seen on MRI, the lesion appearing slightly hyperintense to muscle on T1-weighted images and of intermediate-to-high signal on fluid-sensitive fat saturated sequences (Fig. 5a, b). No extra-osseous tumor was seen; clump-like anterior and small punctate foci of low signal were seen at the sites of ossification seen on radiographs (Fig. 5c–e). Homogeneous enhancement was identified following contrast administration (Fig. 5e). An FDG PET scan showed prominent radionuclide uptake with an SUVmax of 10.9: the CT component of the study showed the ossification seen on radiographs and confirmed severe cortical thinning with a small breach of the anterior cortex of the ulna (Fig. 4c). No other lesions were identified. The imaging appearances were considered nonspecific, but in view of the internal ossification, a bone-forming tumor with some locally aggressive features (for example, low-grade central osteosarcoma) was considered: as in case 1, no marrow edema-like signal was identified to suggest osteoblastoma.

Preoperative testing for disorders of calcium and phosphate was not performed: there were no clinical or radiographic features to suggest tumor-induced osteomalacia.

**Fig. 6:**
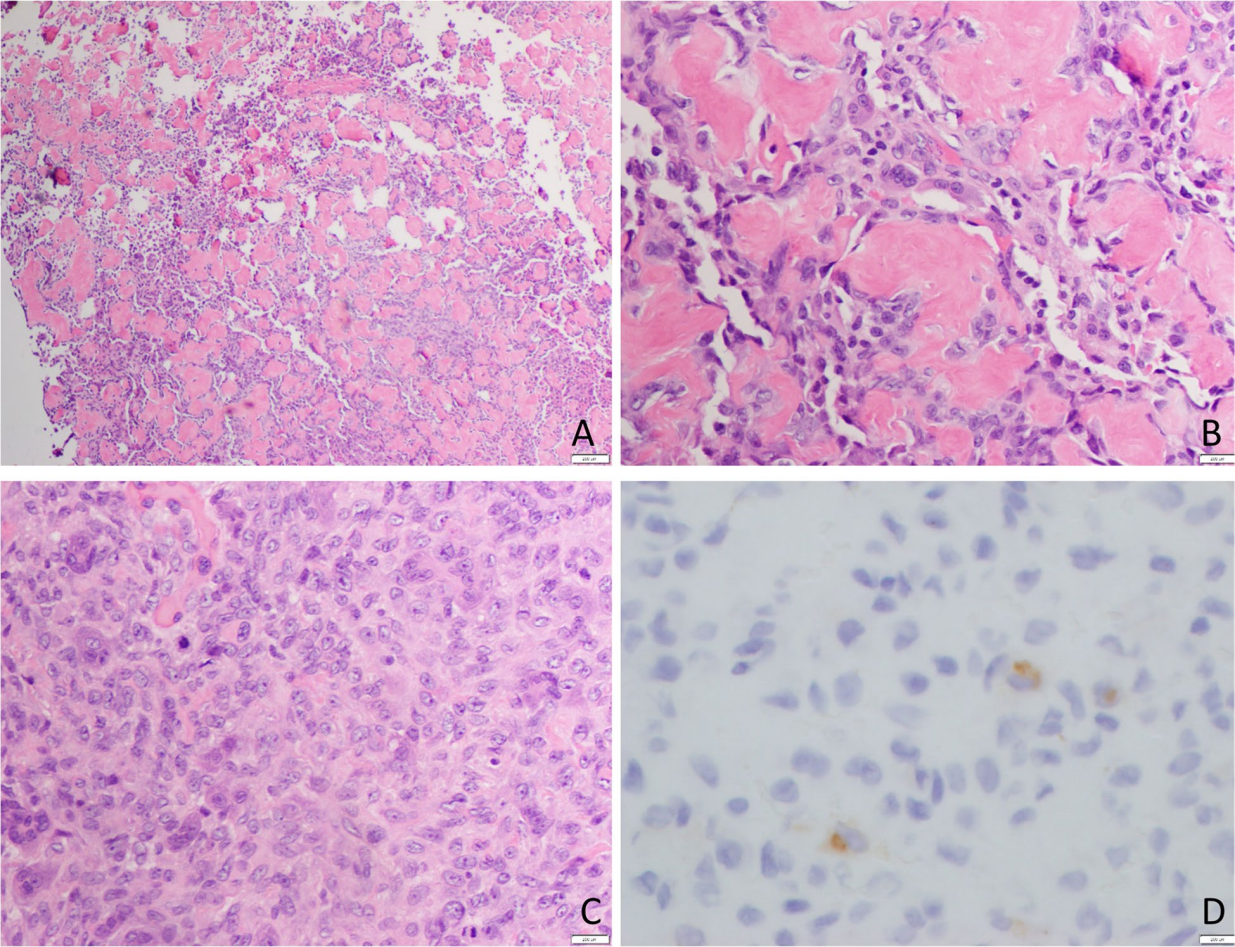
Bone forming tumor with an osteoblastoma-like appearance and focal tendency to cellular discohesion. At higher power, there are nodular areas of immature osteoid rimmed by mononuclear cells with admixed osteoclast giant cells (seen in **B**). A clearly vascularized stroma is not seen. The mononuclear cells lack the usual eccentric nuclei and paranuclear clearing usually seen in osteoblastoma (**A**, **B**). In more solid areas devoid of osteoid, the cells show a tendency to spindling and streaming. They have vesicular nuclei and small prominent nucleoli. Admixed small osteoclastic giant cells are noted (**C**). Immunostaining shows FGF23 paranuclear dot-like expression in occasional cells only (**D**)

Core biopsy was followed by intralesional excision. Via a posterolateral corticotomy, the lesion was carefully curetted, with adjuvant liquid nitrogen and lavage before impaction grafting the defect with allograft bone. A bone-forming tumor was identified, with rounded and focally anastomosing trabeculae of woven bone, lined by plump osteoblastic cells in a background stroma containing dilated vascular channels, vacuolated histiocytic cells, and admixed giant cells. Focally, cell discohesion was noted (Fig. 6). In areas, a tendency to spindling was noted, giving a “fibrohistiocytic” appearance. Bone formation was lacking in some regions; nuclear indentation, with overlapping nuclei and multinucleation, was noted. Nuclear variability and mild atypia were also seen, with some prominent nucleoli. However, frank anaplasia was lacking, and mitoses were sparse overall, reaching 4 per mm^2^ in occasional areas. Atypical mitoses were not noted; necrosis was not seen, and there was no evidence of host bone permeation. Some fragments of the lesion had a well-defined edge abutting fibrous tissue.

H3.3 G34W expression was not identified and there was no evidence of *MDM2* gene amplification. Due to the unusual nature of this lesion, next generation RNA sequencing was performed and identified an *NIPBL::BEND2* fusion. No additional mutations were noted on DNA sequencing. SNP array showed a “quiet”/diploid genome.

Following identification of the fusion, immunostaining of the tumor showed expression of CD56 and SSTRA2 in most cells (common in phosphaturic mesenchymal tumor) [4] and an occasional cell showed paranuclear dot-like expression of FGF23. However, in the absence of clinical, radiographic, or morphological evidence of PMT, this case was best characterized as an atypical, bone-forming osteoblastoma-like tumor, the biological behavior of which could not be predicted. Methylome profiling (Infinium Human Methylation Bead Chip Array) was also performed and showed similarities to both osteoblastoma and PMT, but neither was a clear match. There was focal gain of chromosome 4p, but there was an otherwise flat copy number profile.

The patient has remained well following her recent surgery.

## Discussion

These cases extend the limited but heterogeneous clinical and pathological data previously reported on bone-forming tumors driven by a *NIPBL::BEND2* fusion. Two of thepublished cases with this fusion were classified as PMT: the lesion in the fibula [1] was described as osteoblastoma-like, whereas little information was provided about the histological appearance of the second lesion, a 6 cm tumor in the ilium of a 31-year-old male [2]. One high-grade malignant bone-forming tumor demonstrating this fusion has also been reported, most closely resembling an osteosarcoma [3]. On DNA methylation profiling, this tumor clustered with high-grade osteosarcoma, rather than PMT. Histologically, our first case resembled an osteosarcoma (with no high-grade features and no relapse to date), and the second a low-grade, bone-forming (osteoblastoma-like) tumor with no histological features to suggest PMT. Following identification of the *NIPBL::BEND2* fusion; however, expression of CD56 and SSTRA2 and occasional paranuclear dot-like expression of FGF23 were identified immunohistochemically, suggesting some overlap with PMT. The methylation profile of this lesion also showed similarities to both PMT and osteoblastoma. The future biological behavior of both lesions is unpredictable, with follow-up longer in the first case: notably, the previously reported metatarsal bone-forming lesion [3] showed initially indolent but ultimately locally aggressive behavior. Unfortunately, serum FGF23 was not measured in either of our cases, but there were no clinical, biochemical, or radiographic features of tumor-induced osteomalacia.

As with the pathology, the imaging appearances of the published cases are also heterogeneous. The fibular lesion [1] showed predominantly ground-glass density with possible small punctate foci of mineralization. Prominent, apparently circumferential intra-cortical growth was seen. No imaging of the iliac tumor [2] is available. The metatarsal lesion [3] was initially intramedullary, mildly expansile and showed lytic destruction, with dense matrix ossification in the proximal aspect of the tumor but no intra-cortical growth. It eventually recurred with a densely ossified extra-osseous mass which destroyed adjacent metatarsals and cuneiforms. PMTs can occur in bone and soft tissue. The imaging features of the bone lesions are variable: mineralization is common (amorphous, punctate or ground glass [5]), but dense ossification and intra-cortical growth are infrequent. Both cases in this report showed radiographic evidence of tumor ossification, diffusely in the radius and in a small punctate foci in the ulna.

*NIPBL* (Nipped-B-like: the human homolog of Nipped-B in Drosophila) encodes for a protein that facilitates the loading of cohesin onto chromosomes, enabling DNA repair and transcription [6]. Mutations in *NIPBL* cause Cornelia de Lange syndrome [7], which affects multiple systems and is associated with a predisposition to tumors, most commonly esophageal adenocarcinoma (typically associated with Barrett’s esophagus), and occasionally other tumors (Wilm’s tumor, leukemia, endometrial carcinoma, and pancreatic neuroendocrine tumors [8]). Somatic mutations in *NIPBL* have been found in gastric and colorectal cancers [9] and fusions have been described in intrahepatic cholangiocarcinoma [10], acute megakaryoblastic leukemia [11] and atypical tenosynovial giant cell tumor [12].

*BEND2* (BEN Domain Containing 2) encodes one of several BEN domain-containing proteins which influence transcription and remodeling of chromosomes by recruitment of chromatin modifying factors [13]: it is an essential regulator of meiosis during spermatogenesis in mice [14]. Fusions involving *BEND2* are seen in astroblastoma-like central nervous system tumors, characteristically associated with *MN1* alterations [15], but also with other fusion partners (*EWSR1* [16], *MAMLD1* [17], *YAP1* [18]). Fusion with *TCF3* [19] is associated with intracranial neuroepithelial tumors. *BEND2* fusions are also associated with neuroendocrine tumors in the pancreas [20] and a soft tissue sarcoma of the abdominal wall [21] was found to harbor an *MN1::BEND2* fusion, previously considered to define astroblastoma.

The role of the *NIPBL::BEND2* fusion in the etiology of bone tumors is uncertain: transfected cells showed enhanced proliferation at 48 h and upregulation of MYC-target genes, suggested to be due to the fusion [1].

In conclusion, based on the limited information provided by the previous literature and this report, bone lesions with *NIPBL::BEND2* fusion should be considered in the differential diagnosis of bone-forming tumors. A single published case [3] has demonstrated histological features of high-grade osteosarcoma; our cases more closely resembled low-grade lesions, indicating that bone-forming tumors of different grades are possible. Our second case showed histological features of a locally aggressive bone-forming (osteoblastoma-like) tumor but also subtle immunohistochemical features suggestive of PMT. Methylome profiling showed similarities to both entities without a clear match. The nature of these unusual lesions cannot currently be predicted and requires evaluation of further cases with longer follow-up.

## Data Availability

Not applicable.
